# Perforated sigmoid diverticulitis in a lumbar hernia after iliac crest bone graft - a case report

**DOI:** 10.1186/1471-2482-14-46

**Published:** 2014-07-22

**Authors:** Florian S Frueh, Raphael N Vuille-dit-Bille, Dimitri A Raptis, Hanspeter Notter, Brigitte S Muff

**Affiliations:** 1 Department of Surgery, Hospital of Bulach, Spitalstrasse 24, 8180 Bulach, Switzerland; 2 Division of Plastic Surgery and Hand Surgery, University Hospital of Zurich, Zurich, Switzerland; 3 Department of Pediatric Surgery, University Children’s Hospital of Zurich, Zurich, Switzerland; 4 Department of Visceral and Transplantation Surgery, University Hospital Zurich, Zurich, Switzerland

**Keywords:** Perforated diverticulitis, Lumbar hernia, Iliac crest bone graft, Laparoscopic hernia repair, Mesh

## Abstract

**Background:**

The combination of perforated diverticulitis in a lumbar hernia constitutes an extremely rare condition.

**Case presentation:**

We report a case of a 66 year old Caucasian woman presenting with perforated sigmoid diverticulitis localized in a lumbar hernia following iliac crest bone graft performed 18 years ago. Emergency treatment consisted of laparoscopic peritoneal lavage. Elective sigmoid resection was scheduled four months later. At the same time a laparoscopic hernia repair with a biologic mesh graft was performed.

**Conclusion:**

This case shows a very seldom clinical presentation of lumbar hernia. Secondary colonic resection and concurrent hernia repair with a biologic implant have proven useful in treating this rare condition.

## Background

Complicated diverticulitis may be classified according to Hinchey into four stages [1] (Table 1). Treatment of perforated diverticulitis with peritonitis is a source of controversy [2]. Patients with purulent or fecal peritonitis corresponding to Hinchey III and IV require Hartmann’s procedure (i.e. immediate resection of the diseased segment with end colostomy followed by secondary reversal) or colonic resection with primary anastomosis, with or without protective ileostomy [3]. Laparoscopic peritoneal lavage with secondary colonic resection and anastomosis reflects a treatment option of diverticulitis with pelvic or retroperitoneal abscess formation not amenable to percutaneous drainage, but recently it was also employed to treat Hinchey III disease [2,4].

**Table 1:** **Hinchey classification of diverticulitis**[1]

	
Stage I	Pericolic abscess or phlegmon
Stage II	Pelvic, intra-abdominal or retroperitoneal abscess
Stage III	Generalized purulent peritonitis
Stage IV	Fecal peritonitis

Lumbar hernias and especially those following iliac crest bone graft are extremely rare. Only about 300 cases have been reported in the literature [5]. Strategies of surgical lumbar hernia repair include primary repair, local tissue flaps, open mesh repair or laparoscopic mesh repair [6]. None of these methods has yet been defined or accepted universally.

Here, we describe an extremely rare combination of perforated diverticulitis in a lumbar hernia.

## Case presentation

**Figure 1:**
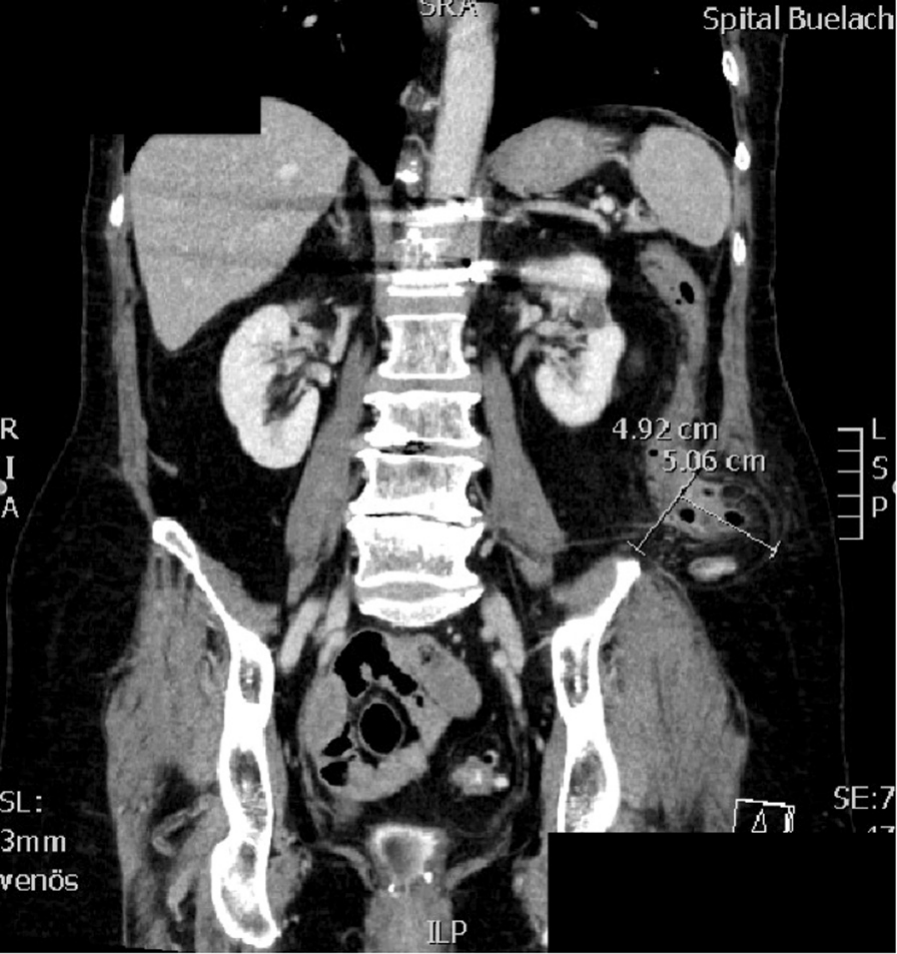
Abdominal CT scan, showing perforated sigmoid colon in a left lumbar hernia.

**Figure 2:**
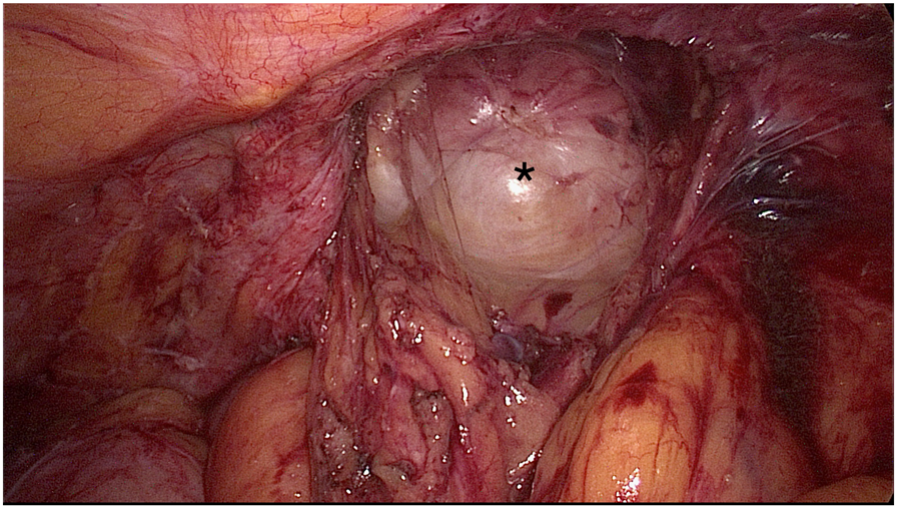
Left lumbar hernia (measuring 5×5 cm) marked with a star (*).

**Figure 3:**
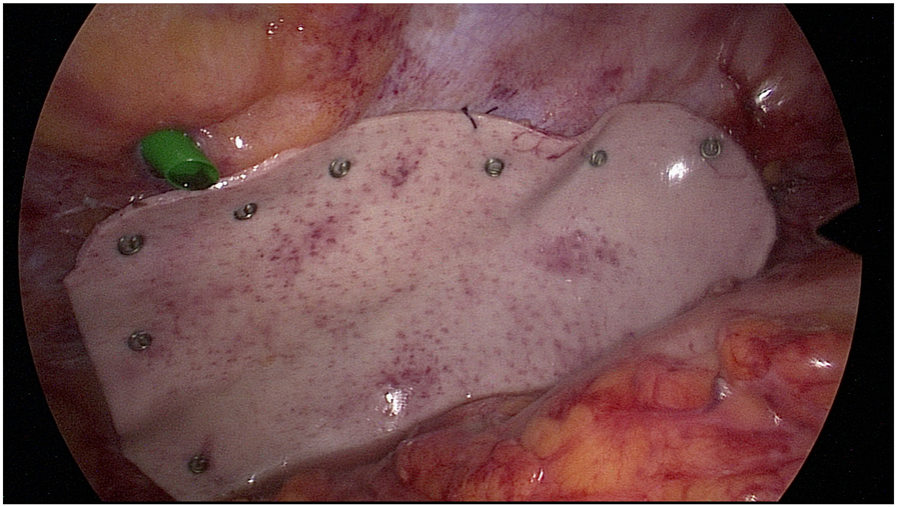
**Permacol**^**® **^**mesh fixation.**

A 66 year-old female Caucasian patient (BMI 26.4 kg/m^2^) presented with a one-week history of lower left abdominal pain. Her general practitioner had already introduced empirical antibiotics (Metronidazole and Ciprofloxacin) five days before presentation based on a suspicion of diverticulitis. The patient’s condition rapidly deteriorated after initial improvement under antibiotic therapy. Eighteen years previously, the patient had spinal fusion of the thoracolumbar transition with bone graft from the left iliac crest due to vertebral body fracture. Since then she complained of a mass on her left flank. In addition, the patient was known to suffer from hypertension, dyslipidemia, hypothyroidism and osteopenia. Physical examination exhibited tenderness in the left lower quadrant, normal bowel sounds and a bulge in the left flank. Reduction of the hernia was not possible due to pain. Blood laboratory values showed elevated C-reactive protein (41 mg/dl) and a white blood cell count within normal range. The patient’s temperature was 37.2°C. A CT scan was performed showing diverticulitis of the sigmoid colon with free perforation, herniating partially in a left lumbar hernia, which we classified as Petit’s hernia based on its localization (Figure 1). There were no signs of strangulation. Immediate laparoscopic hernia reduction was performed. We observed a small amount of purulent fluid in the region of the cranial part of the hernia orifice, as well as fibrin overlying the sigmoid colon. We did not identify a patent communication between the colonic lumen and the peritoneal cavity as the inflammatory process had sealed the original perforation. No pus or stool was seen in the remaining abdomen. According to the Hinchey classification for acute diverticulitis [7] due to the presence of free intraabdominal air together with a local putrid peritonitis, the present case was rated as stage III disease. Hence, peritoneal lavage with 12 liters of warm Ringer’s solution was performed. The operation time was 90 minutes and no intraoperative complications occurred. An intraperitoneal drainage was inserted during the operation and was removed four days after surgery. The antibiotics were continued for 15 days. The patient’s postoperative hospital stay was without any complications and she was discharged eight days after surgery.Seventeen weeks later the patient was admitted for elective laparoscopic sigmoid resection in combination with hernia repair using a biologic mesh (Permacol®, crosslinked porcine collagen, Covidien, Mansfield MA, USA). General anesthesia was introduced and the patient was placed in supine position that was not changed during the procedure. A ureteral catheter was placed to visualize the left ureter. Carbone dioxide insufflation and introduction of a 50°-laparoscope (Karl Storz GmbH & Co, Tuttlingen, Germany) was performed above the umbilicus in order to ensure a good overview. Two 12 mm-trocars were introduced in the right lumbar region and above the symphysis, respectively. The forth trocar (5 mm) was placed in the left hypochondrium. Adhesions between the descending and sigmoid colon were divided with the Harmonic® scalpel (Johnson & Johnson AG, Zug, Switzerland). The left colonic flexure was completely mobilized in order to better visualize the hernial orifice. The defect in the abdominal wall measured approximately 5 cm in diameter and lied within close proximity to the left iliac crest (Figure 2). A Permacol® mesh measuring 10×15 cm was inserted and fixation of the mesh was performed with 5 mm helical titanium tacks (ProTack®, Covidien, Dublin, Ireland, Figure 3). Subsequently, a laparoscopic segmental sigmoid resection with end-to-end double-staple anastomosis using an Endo GIA™ Universal Stapling System (Covidien, Dublin, Ireland) was performed. Despite the higher risk of bacterial contamination of the implant, the mesh fixation was performed prior to anastomosis in order to have a better overview and to minimize mobilization of the freshly anastomosed sigmoid colon. No intra- or postoperative complications occurred and the patient was discharged on the eighth postoperative day. Clinical follow-up appointments were performed after three and 14 months, without any signs of hernia recurrence or digestive complaints.

## Discussion

### Lumbar hernias

Lumbar hernias may arise in the superior and inferior lumbar triangle. The superior lumbar triangle hernia, first described by Grynfeltt and so named as Grynfeltt’s hernia, is bordered by the 12^th^ rib, the erector spinae muscle and the internal oblique muscle. Similarly, the inferior lumbar triangle hernia is referred to as Petit’s hernia and is bordered by the latissimus dorsi muscle, the iliac crest and the external oblique muscle [5,6,8]. Both types of hernias may be divided into congenital and acquired lumbar hernias with the latter occurring after full-thickness iliac crest harvest or following nephrectomy. Risk factors for lumbar hernias include advanced age, female gender, obesity, and poorly developed abdominal muscles [9]. Similarly to other abdominal wall hernias, optimal treatment of lumbar hernias requires surgical repair. But the approach to the posterior abdominal wall defect is challenging and the absence of a musculoaponeurotic layer, the weak posterior abdominal wall muscles and the limiting bony structures complicate dissection and fixation of a mesh [5]. Different methods for hernia closure have been described including primary closure, rotational and onlay fascial flaps, prosthetic material, or straightening the iliac crest [6]. Whereas primary closure is often inadequate for lumbar hernia repair due to high tension in the suture, rotational flaps need extensive tissue dissections and bear the risk of compromised vascularization. Prosthetic mesh placement displays a safe and effective method reducing the tension especially where there is significant separation of the margins. Open surgery brings the drawback of a large incision to clearly define the defect. A laparoscopic approach seems to be favorable due to less postoperative pain and earlier hospital discharge. For laparoscopic repair the literature recommends a semi-prone position with 45° elevation of the affected side in order to improve exposure by placing the intestine to the reverse side pursuant to gravity. Placement of trocars is endorsed subxiphoidly, periumbilically and suprapubicly [5]. In the present case, the surgeon chose supine patient positioning allowing mesh placement and sigmoid resection with adequate overview without changing the position of the patient.

### Perforated diverticulitis

Standard procedures for purulent or fecal peritonitis (Hinchey III and IV) consist of Hartmann’s procedure or colonic resection with primary anastomosis in the acute phase, with or without protective ileostomy formation [3]. Outcomes are amendable with about 10 per cent of patients showing stoma-related complications following Hartmann’s procedure and up to 14 per cent of patients showing anastomotic leakages following primary anastomosis [2]. Furthermore, the stoma is never reversed in about two third of patients following Hartmann’s procedure [10].

Emergency laparoscopic lavage followed by colonic resection and anastomosis in a second procedure represents a relatively new strategy in the management of perforated diverticular disease. It was initially recommended for selected patients with Hinchey I and II perforated diverticulitis [11]. More recently, several authors have reported laparoscopic lavage as a treatment option also for Hinchey III disease [2] providing adequate control of the acute inflammatory episode in most patients. Of course, laparoscopic lavage is to be reserved for patients with purulent peritonitis and abscesses not accessible to percutaneous image-guided drainage. Nevertheless, the Hinchey classification refers to intra-operative findings. Hence one advantage of laparoscopy is exploration of the peritoneal cavity in order to categorize patients. A further advantage is the relatively short operation time [10,11].

### Combination of perforated diverticulitis and lumbar hernia

The described clinical presentation represents an extremely rare finding. If the patient did not have a lumbar hernia and was suffering from perforated diverticulitis only, a laparoscopic drainage would have been performed due to free bowel perforation as reported in the CT scan. As laparoscopic mesh repair is the recommended treatment option for secondary lumbar hernia repair by many authors [5,6,8,9], we decided to combine these two procedures. Of course, it is imperative that implanting foreign material, even biological, should be avoided in an acute infection. Hence, laparoscopic hernia reduction was combined with laparoscopic peritoneal lavage and mesh repair was performed simultaneously with secondary colonic resection and anastomosis. The advantage of this “two-step” procedure is the avoidance of a third general anesthesia (including possible peri- and postoperative complications), but of course, the combination of elective colorectal surgery and mesh implantation brings the risk of foreign material contamination with colonic bacteria. Whereas some authors found that concomitant mesh repair and colorectal surgery showed reasonable outcomes in most patients [12,13], others stated a clearly increased risk of infectious and noninfectious complications [14] and requested a critical case-by-case patient selection for simultaneous surgery. According to the decision model for biologics by the Italian Biological Prosthesis Work-Group (IBPWG), a high resistance to mechanical stress and to protease enzyme action favors the use of cross-linked biological prostheses in circumstances of high infection probability and/or large tissue defects [15]. Due to the high possibility of bacterial contamination upon concomitant colorectal surgery and because of the size of the defect (5x5 cm) a cross-linked biological prosthesis was chosen for the present patient.

## Conclusions

If perforated, diverticulitis may be treated by laparoscopic lavage (i.e. not in Hinchey IV disease). Concurrent laparoscopic hernia reduction displays a feasible treatment option. Secondary colonic resection and primary anastomosis in the infection-free interval may be combined with the closure of the hernia defect. Laparoscopic mesh implantation may exhibit the most convenient method for lumbar hernia repair, but the surgeon should avoid mesh repair in the infectious state and should be careful with simultaneous elective colorectal surgery and foreign material implantation.

### Consent

Informed consent was obtained from the patient for publication of this case report and all images.

## Abbreviations

BMI: Body mass index.

## Competing interest

The authors declare that they have no competing interest.

## Authors’ contributions

FSF and HN performed the data acquisition (including patient’s information and informed consent). FSF, RNV, DAR, HN and BSM participated in the design and coordination of this case report. FSF, RNV and DAR drafted the manuscript. HN and BSM helped to draft the manuscript. All authors read and approved the final manuscript.

## Pre-publication history

The pre-publication history for this paper can be accessed here:

http://www.biomedcentral.com/1471-2482/14/46/prepub
